# Preoperative short-course radiotherapy followed by consolidation chemotherapy for treatment with locally advanced rectal cancer: a meta-analysis

**DOI:** 10.1186/s13014-021-01974-4

**Published:** 2022-01-24

**Authors:** Haoyan Wu, Chuanwen Fan, Chao Fang, Libin Huang, Yuan Li, Zongguang Zhou

**Affiliations:** 1 Laboratory of Digestive Surgery, State Key Laboratory of Biotherapy and Cancer Center, Sichuan University, Chengdu, 610041 China; 2 Department of Gastrointestinal Surgery, West China Hospital, West China School of Medicine, Sichuan University, No. 37, Guo Xue Xiang, Chengdu, 610041 China; 3 Department of Pediatric Surgery, West China Hospital, West China School of Medicine, Sichuan University, Chengdu, 610041 China; 4 Department of Gastrointestinal Surgery and Breast and Thyroid Surgery, Minimally Invasive Surgery, West China School of Public Health, Sichuan University, Chengdu, 610041 China; 5 West China Fourth Hospital, Sichuan University, Chengdu, 610041 China; 6 Department of Oncology, Linköping University, 58183 Linköping, Sweden; 7 Department of Biomedical and Clinical Sciences, Linköping University, 58183 Linköping, Sweden

**Keywords:** Rectal cancer, Short-course radiotherapy, Consolidation chemotherapy, Meta-analysis

## Abstract

**Background:**

The addition of consolidation chemotherapy to preoperative short-course radiotherapy during the prolonged interval between the completion of radiation and surgery in locally advanced rectal cancer (LARC) could enhance pathologic response and might act on potential micrometastasis. We performed this meta-analysis to evaluate whether short-course radiotherapy followed by consolidation chemotherapy (SCRT/CCT) could be a neoadjuvant treatment option compared with conventional long-course chemoradiotherapy (LCCRT).

**Methods:**

We searched the PubMed, EMBASE, MEDLINE, and Cochrane Library databases. The primary endpoints were pathological outcomes, and the secondary endpoints included survival rate, sphincter preservation rate, R0 resection rate and toxicity. RevMan 5.3 was used to calculate pooled risk ratio (RRs) and 95% confidence intervals (CIs).

**Results:**

A total of seven eligible studies and 1865 participants were included in this meta-analysis. Compared with the LCCRT, SCRT/CCT increased pathologic complete response (pCR) rate [RR = 1.74, 95% CI (1.41, 2.15), P < 0.01] and led to a lower proportion of patients with adjuvant pathologic tumor stage 3–4 (ypT3-4) disease [RR = 0.88, 95% CI (0.80, 0.97), P = 0.01] or lymph node positive (ypN +) disease [RR = 0.83, 95% CI (0.71, 0.98), P = 0.02]. In addition, the disease-free survival (DFS) was better in SCRT/CCT group [RR = 1.10, 95% CI (1.02, 1.18), P = 0.01], while overall survival rate and toxicity and surgical procedures were similar between two groups.

**Conclusion:**

Based on better pathological outcomes and DFS in SCRT/CCT group, we recommended preoperative short-course radiotherapy followed by consolidation chemotherapy as the optional neoadjuvant treatment for LARC.

**Supplementary Information:**

The online version contains supplementary material available at 10.1186/s13014-021-01974-4.

## Background

There are two general approaches to preoperative neoadjuvant treatment for locally advanced rectal cancer (LARC). Conventionally chemoradiotherapy (CRT), consisting of long-course radiotherapy with concomitant fluoropyrimidine chemotherapy, is the current standard treatment for TNM stage II and III rectal cancer in the United States and southern Europe, whereas short-course radiotherapy (SCRT) with immediate surgery is more commonly applied in the north Europe [1–3]. Both preoperative neoadjuvant treatments, with similar safety and efficacy, have decreased local recurrence and improved survival rate [4–6]. The distant disease recurrence, however, has not decreased accordingly and remained a substantial problem.

SCRT with immediate surgery, which is advised for those with intermediate risk rectal cancer or contraindications to long-course radiation, is inferior to conventional CRT in terms of pathologic complete response (pCR) rate and tumor downstaging. Desired pathological outcomes occur when surgery is delayed after SCRT, as found in the Stockholm III trial and a systematic review in 2014 [3, 7, 8], which both addressed the optimal interval between the completion of radiotherapy and resection of tumor. The interval could be prolonged appropriately, creating an opportunity to deliver systemic chemotherapy preoperatively, which, to some extent, might act on obscure micrometastases and thereby reduce distant metastasis [9]. Some centers have administered postoperative adjuvant chemotherapy with the intention of reducing distant failure, but the effect and compliance were far from satisfactory [10]. However, the upfront systemic chemotherapy, which is delivered in the waiting period between SCRT and resection, is well-tolerated, as reported by several trials and the cooperation regimen of consolidation chemotherapy and delayed surgery after SCRT had high neoadjuvant therapy completion rate and tumor downstaging [11–14].

Based on these findings, it is concluded that such a combination of the prolonged waiting period between the completion of SCRT and resection and delivering consolidation chemotherapy during the interval might be superior to conventional long-course chemoradiation. Herein, we report a meta-analysis of all those published studies adopting the short-course radiotherapy followed by consolidation chemotherapy (SCRT/CCT) for LARC with the aim of comparing pathological outcomes and survival rates to those of conventional long-course chemoradiotherapy (LCCRT).

## Material and methods

### Inclusion criteria

According to the PICOS principles, we defined the following inclusion criteria: (1) Participants (P): studies involving patients with nonmetastatic rectal cancer confirmed by biopsy and receiving neoadjuvant treatment. (2) Interventions (I) and comparisons (C): studies comparing SCRT/CCT with LCCRT as neoadjuvant treatment in LARC. The SCRT/CCT regimen was 25 Gy in five fractions, regardless the use of concurrent chemotherapy, followed by several cycles of consolidation chemotherapy before surgery; the LCCRT regimen was long-course radiotherapy with concomitant chemotherapy followed by surgery. (3) Outcomes (O): studies evaluating following outcomes: pCR rate, adjuvant therapy pathologic stage(ypTNM), local recurrence (LR), distant metastasis (DM), overall survival rate (OS), disease free survival rate (DFS), sphincter preservation rate, radical (R0) resection rate, postoperative complications, downstaging rate, acute toxicity and late complications. (4) Study design (S): prospective and retrospective studies.

We excluded the following publications: (1) studies involving patients with synchronous metastases or serious cardiopulmonary diseases or other severe basic diseases; (2) short-course radiotherapy without consolidation chemotherapy before surgery; (3) studies were not controlled trials, for example, single arm study, case series or case report; (4) studies lacking complete important information for extracting the required data; and (5) non-original studies, such as letters, reviews, and expert opinions.

### Literature search

We systematically searched the PubMed, MEDLINE, EMBASE, and the Cochrane Controlled Trials Register by using terms of “short-course radiotherapy”, “chemotherapy”, “long-course chemoradiotherapy”, Medical Subject Heading (MeSH) terms “rectal Neoplasms” and its individual corresponding free terms with combination of Boolean operators (AND, OR, NOT). There was no language restriction. The last search was updated on 21 July, 2021. In addition, we reviewed references in the retrieved articles to search for additional relevant studies.

### Assessing risk of bias of included studies

The quality of randomized controlled trials (RCTs) was assessed using the Cochrane Collaboration’s risk for bias assessment tool [15], which evaluated the selection bias, performance bias, detection bias, attrition bias, and reporting bias. The quality of cohort studies was measured by a score system assessed in accordance with the Newcastle–Ottawa criteria [16]. The total scores ranged from 0(worst) to 9(best) for cohort studies, with a score of at least 6 indicating high quality. Each criterion was assessed as low risk for bias, high risk for bias, or uncertain risk for bias.

### Data extract

The following information were extracted from each selected paper if available: first author, year of publication, number of patients, type of study, follow-up time, intervention and comparison, OS, DFS, LC, DM, sphincter preservation rate, R0 resection rate, postoperative complications, pCR rate, downstaging rate, ypTNM stage, acute toxicity and late complications.

### Data analysis

All statistical analyses were performed using RevMan 5.3 software. Count data using risk ratio (RR), and 95% confidence intervals (CI) was calculated. Heterogeneity was assumed by using the I^2^ method with the χ^2^ test to calculate P values. If heterogeneity was not present (P > 0.10, I^2^ < 50%), a fixed-effect model was adopted for analysis, otherwise, a random-effect will be employed.

## Results

### Study selection

**Fig. 1:**
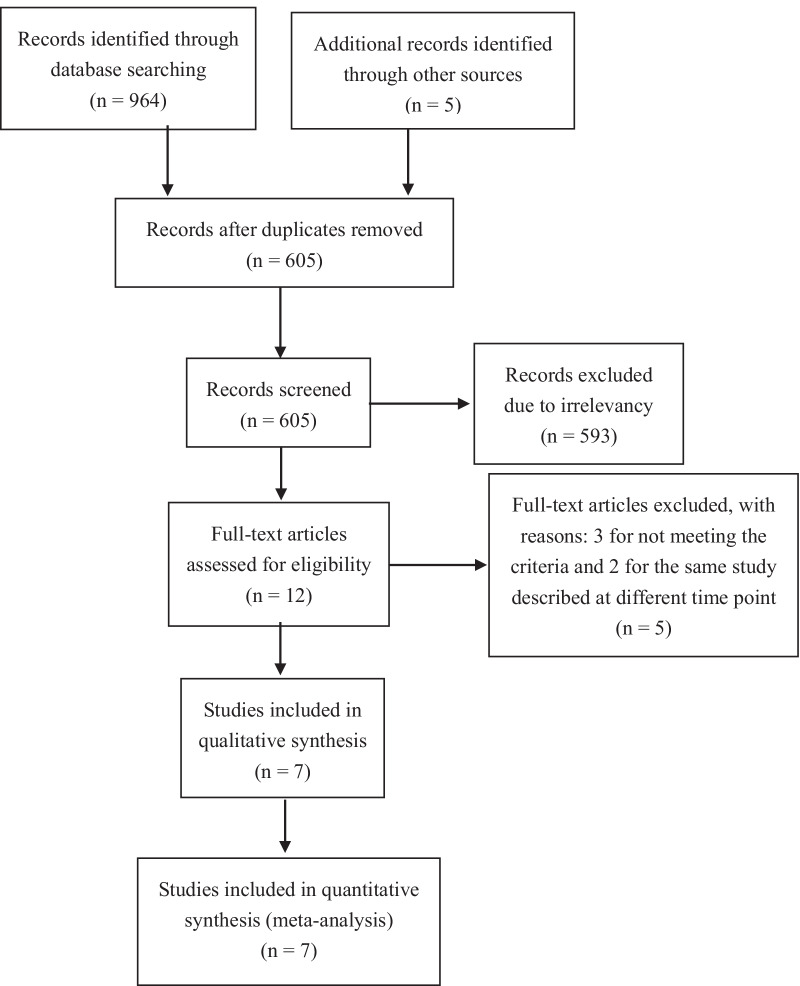
PRISMA 2009 flow diagram

A total of 969 relevant articles were searched, and 364 duplicates were removed. After reviewing the titles and abstracts, 593 of the studies were excluded due to irrelevant. Next, 12 potential eligible full-text articles were further evaluated. We excluded another 5 full-text articles, including 3 articles for not meeting the criteria for SCRT/CCT and 2 articles that were the same study described at different time point. Finally, we included seven studies in the meta-analyses (Fig. 1). Four of these studies were RCTs [9, 17–19], and the other three were non-RCTs [20–22].

All of the involved RCTs mentioned that patients were informed of their treatment plan at allocation. However, this limitation was unlikely to affect the results of quality assessment (Additional file 1: Fig. 1 and Additional file 1: Fig. 2, which demonstrated risk of bias graph and risk of bias summary). The three non-RCTs were all cohort studies including two prospective study [20, 22] and one retrospective study [21]. All three cohort studies scored at least 6 based on the Newcastle–Ottawa criteria (Additional file 1: Table 1).

A total of 1865 patients with locally advanced rectal cancer were assigned to the SCRT/CRT group (n = 928) or LCCRT group (n = 937). The characteristics of studies and patients, shown in Table 1 and Additional file 1: Table 2, were similar between the two treatment groups.

**Table 1:** Characteristics of the 7 included studies

References	Study design	Sample size	Intervention		Follow-up time (months)	Outcomes
			SCRT/CCT	LCCRT		
Markovina et al. [20]	Prospective study	138	25 Gy in 5 fractions, 4 cycles FOLFOX, TME	median 45 Gy in 25 fractions with concurrent CT, TME	49.4/54.3	OS, DFS, LRR, DMR, pCR rate, ypTNM, downstaging, sphincter preserve rate, R0 resection rate, toxicity
Chung et al. [21]	Retrospective study	72	25 Gy in 5 fractions with concurrent CT, 3 cycles CT, TME	50.4 Gy in 28 fractions with concurrent CT, TME	25	OS, DFS, LRR, DMR, pCR rate, downstaging, sphincter preserve rate, R0 resection rate, toxicity
Ciseł et al. [17]	Randomized controlled trials	515	25 Gy in 5 fractions, 3 cycles FOLFOX4, TME	50.4 Gy in 28 fractions with concurrent CT, TME	84.0	OS, DFS, LRR, DMR, pCR rate, ypTNM, sphincter preserve rate, R0 resection rate, toxicity
Bahadoer et al. [9]	Randomized controlled trials	912	25 Gy in 5 fractions, 6 cycles CAPOX or 9 cycles FOLFOX, TME	50–50.4 Gy in 25–28 fractions with concomitant CT, TME	55.2	OS, DFS, LRR, DMR, pCR rate, ypTNM, sphincter preserve rate, R0 resection rate, toxicity
Thakur et al. [22]	Prospective study	28	25 Gy in 5 fractions, 2 cycles CT, TME	45 Gy in 25 fractions with concurrent CT, TME	22.6	OS, DFS, LRR, DMR, pCR rate, downstaging, sphincter preserve rate, R0 resection rate, toxicity
Aghili et al. [19]	Prospective study	60	25 Gy in 5 fractions with concurrent XELOX, 3–4 weeks XELOX, TME	50–50.4 Gy in 25–28 fractions with concomitant CT, 3–4 weeks XELOX, TME	18	pCR rate, downstaging, sphincter preserve rate, R0 resection rate, toxicity
Chakrabarti et al. [18]	Randomized controlled trials	140	25 Gy in 5 fractions, 2 cycles XELOX, TME	50–50.4 Gy in 25–28 fractions with concomitant CT, TME	N/A	pCR rate, downstaging, sphincter preserve rate, R0 resection rate, toxicity

SCRT/CCT, short-course radiotherapy followed by consolidation chemotherapy; LCCRT, long-course chemoradiotherapy; CT, chemotherapy; TME, total mesorectal excision; OS, overall survival; DFS, disease-free survival; LR: local recurrence; DM, distal metastasis rate; pCR, pathologic complete response; ypTNM, adjuvant pathologic staging; N/A, not available

### Primary endpoint: pathological outcomes

**Fig. 2:**
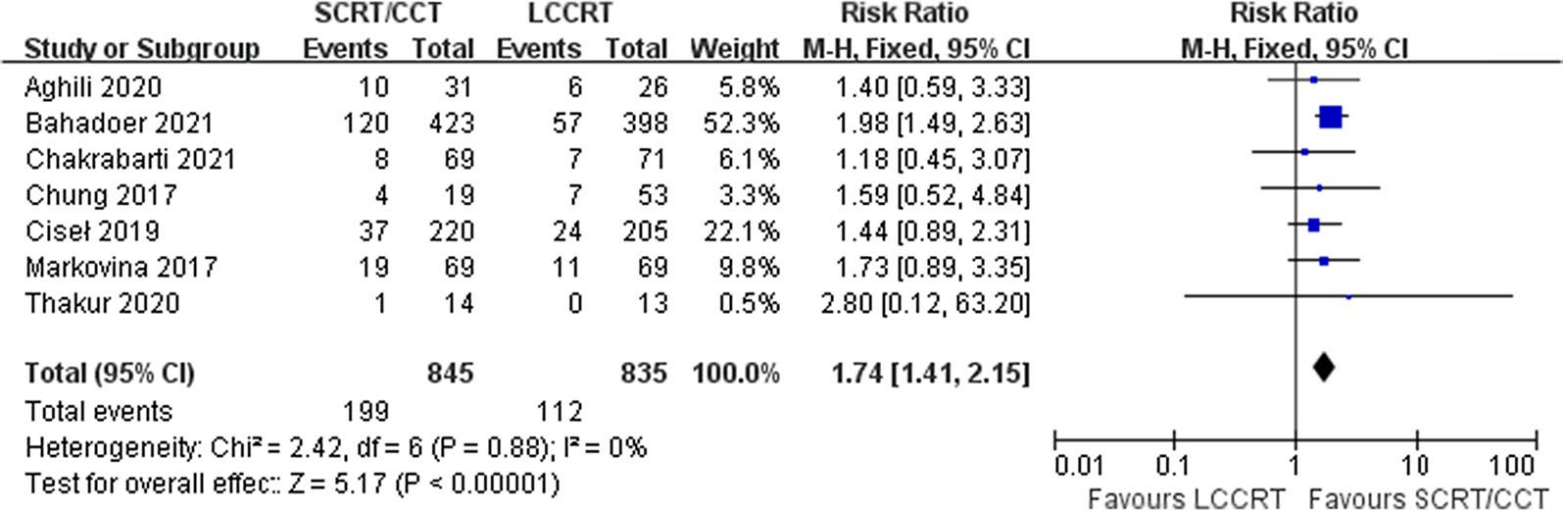
Forest plot for pathological complete response rate

The pathological outcomes consisted of the pCR rate, downstaging rate and ypTNM stage. All seven trials were available for comparative analysis of pCR rate. As shown in Fig. 2, the pCR rate was obviously higher in the SCRT/CCT group [RR = 1.74, 95% CI (1.41, 2.15), P < 0.01; I^2^ = 0%, fixed-effect model]. With respect to downstaging rate, available in five studies involving 434 patients, difference was not significant among recipients of two arms [RR = 1.19, 95% CI (0.86, 1.66), P = 0.30; I^2^ = 78%, random-effect model; Additional file 1: Fig. 3]. Five trials, with a total of 1581 patients, reported ypTNM stage and pooled results suggested that the SCRT/CCT group had a lower proportion of ypT3-4 patients [RR = 0.88, 95% CI (0.80, 0.97), P = 0.01; I^2^ = 31%, fixed-effect model; Additional file 1: Fig. 4] and ypN + patients [RR = 0.83, 95% CI (0.71, 0.98), P = 0.02; I^2^ = 0%, fixed-effect model; Additional file 1: Fig. 5].

### Secondary endpoints: survival rates, toxicity and surgical procedures

The survival events in the involved trials were assessed, when available, at fixed time points (as provided in each study). The median follow-up duration ranged from 22.6 to 84 months.

**Fig. 3:**
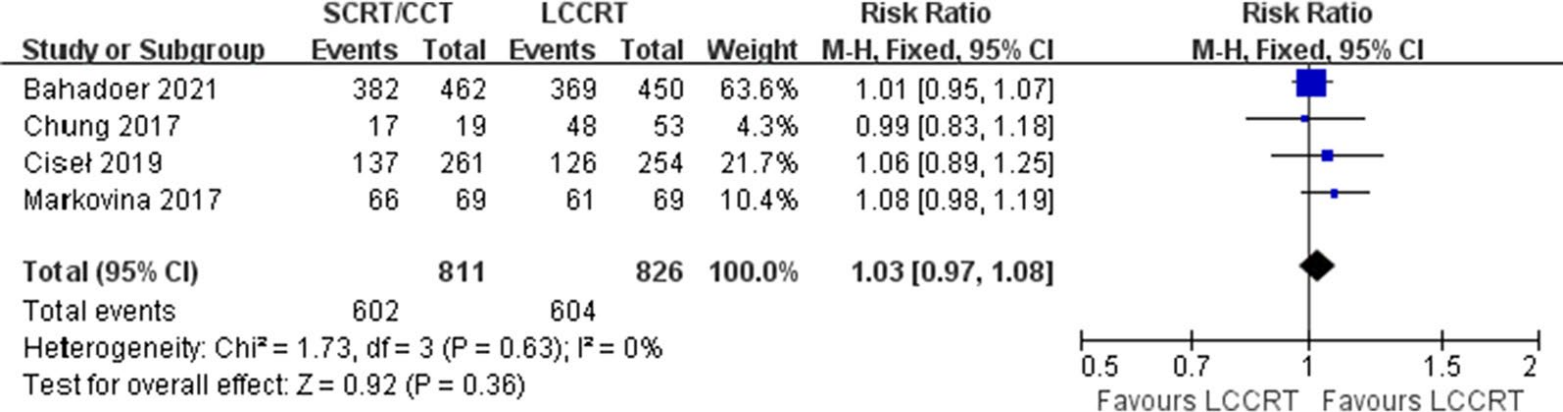
Forest plot for overall survival

**Fig. 4:**
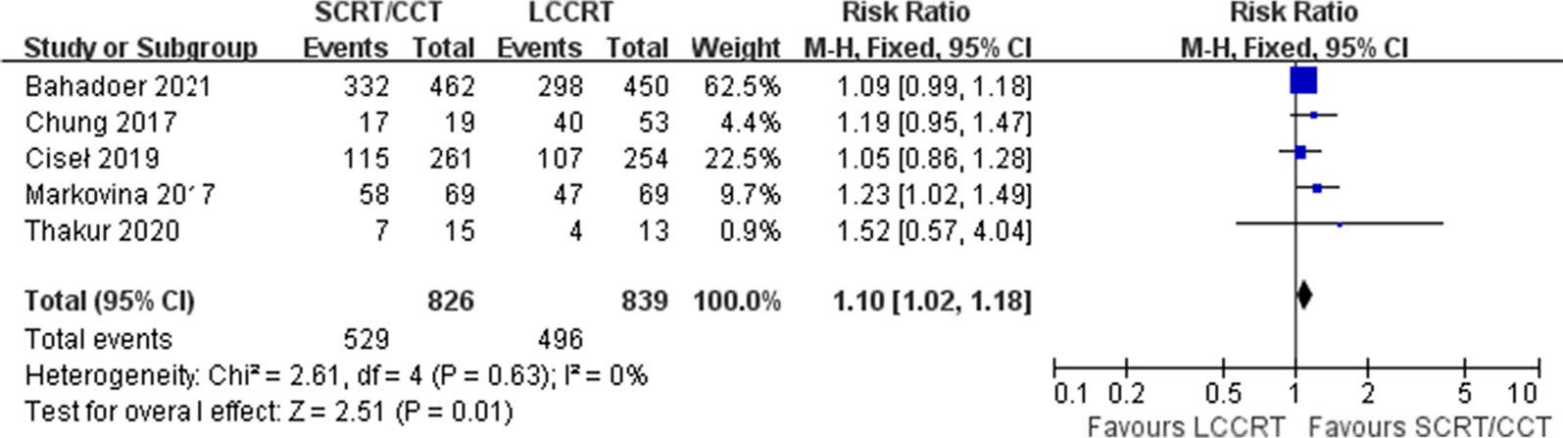
Forest plot for disease-free survival

Survival rates, including OS, DFS, LR and DM, were analyzed. No statistic difference in OS from four available studies was observed between the SCRT/CCT group and LCCRT group [RR = 1.03, 95% CI (0.97, 1.08), P = 0.36; I^2^ = 0%, fixed-effect model; Fig. 3]. DFS, reported in five studies with a total of 1665 patients, was significantly better in the SCRT group [RR = 1.10, 95% CI (1.02, 1.18), P = 0.01; I^2^ = 0%, fixed-effect model; Fig. 4].

LR and DM were reported in five studies. Incidence of LR did not significantly differ between the SCRT/CCT cohort and LCCRT cohort [RR = 1.19, 95% CI (0.95, 1.50), P = 0.13; I^2^ = 0%, fixed-effect model; Additional file 1: Fig. 6]. Despite higher incidence of DM in the SCRT/CCT group, however, the difference did not reach statistical significance [RR = 0.70, 95% CI (0.45, 1.07), P = 0.10; I^2^ = 68%, random-effect model; Additional file 1: Fig. 7].

Toxicity, including acute toxicity, postoperative complications and late complications, was mentioned in all of the included studies. Acute toxicity, which was classified according to the Common Terminology Criteria for Adverse Events and postoperative complications were defined as complications that occurred within 30 days after resection. We assessed only grade three or higher adverse events based on the available data. There were no statistically significant differences between two arms [RR = 1.31, 95% CI (0.92, 1.86), P = 0.13; I^2^ = 66%, random-effect model; Additional file 1: Fig. 8]. RR for post-operation complications and late complications were 1.12 and 1.18, respectively [95% CI (1.00, 1.26), P = 0.06; I^2^ = 0%, fixed-effect model; Additional file 1: Fig. 9] [95% CI (1.00, 1.40), P = 0.05; I^2^ = 29%, fixed-effect model; Additional file 1: Fig. 10], which were close to borderline significance.

The surgical procedures included the R0 resection rate and sphincter preservation rate. R0 resection rate was comparable between the two neoadjuvant treatment groups [RR = 1.04, 95% CI (1.00, 1.09), P = 0.08; I^2^ = 0%, fixed-effect model, Additional file 1: Fig. 11], so was sphincter preservation rate [RR = 1.03, 95% CI (0.93, 1.14), P = 0.57; I^2^ = 0%, fixed-effect model; Additional file 1: Fig. 12].

## Discussion

The meta-analysis demonstrated that the SCRT/CCT group had better DFS and pCR and a lower proportion of ypT3-4 stage patients and ypN + patients than the LCCRT group. The OS and oncological outcomes in the SCRT/CCT group were similar to those in the conventional LCCRT group, as were the toxicity and surgical procedures.

An increased pCR rate and favorable adjuvant pathologic stage were observed in the SCRT group, which could be attributed to delayed surgery and the addition of chemotherapy. Nevertheless, such advantages did not translate to survival benefits or surgical procedures. The prolonged interval between radiotherapy and surgery resulting in pCR benefits has been verified by several randomized trials [7, 23]. The addition of chemotherapy in the SCRT group also likely contributed to improving the tumor response. In a study with four consecutive series of rectal cancer patients receiving 0, 2, 4, or 6 cycles of modified FOLFOX6 after identical chemoradiation before surgery, the pCR rate, compared to that in the chemoradiation alone group, increased by approximately 20% and was as high as 38% in the group delivering six cycles of chemotherapy [24]. Obtaining a pCR, referred to as the eradication of all cancer cells, after preoperative treatment is, to some extent, considered synonymous with a cure. Patients with pCR have been associated with improved survival outcomes, as demonstrated by several pooled studies [25–27]. Additionally, patients with a clinical complete response could follow a watch-and-wait strategy, which is increasingly being used as an alternative to major surgery [28].

DFS is largely influenced by local recurrence and systemic relapse. In our meta-analysis, similar events of LC were observed between the two groups. The occurrence of DM, however, was indeed less common in the SCRT/CCT group, which resulted in better DFS. The differences in DM between the two groups were not statistically significant, as we adopted a random-effect model due to obvious heterogeneity. If a fixed-effect model was applied, the difference would become significant. A meta-analysis suggested that the addition of oxaliplatin to 5FU-based chemoradiotherapy resulted in an increase in pCR and fewer perioperative metastases [29]. In our meta-analysis, almost all patients in the SCRT/CCT group received preoperative chemotherapy containing oxaliplatin. Nevertheless, the reduction in distant metastasis, we suggested, should be attributed to systemic chemotherapy rather than oxaliplatin alone. Improved DFS by consolidation chemotherapy confirmed by a multicenter phase II trial, which evaluated the survival results of LARC patients receiving different cycles of mFOLFOX during the period between chemoradiation and surgery [30]. Compared with that in patients who received only chemoradiation, DFS was better in patients who received additional preoperative chemotherapy. Systemic chemotherapy was also administered in the LCCRT group, but it usually began 6-8 weeks after surgery, which is much later than preoperative chemotherapy. In addition, the rate of adherence to adjuvant chemotherapy is unsatisfactory, as demonstrated by the largest adjuvant trial for LARC (EORTC 22921 study) and one of the included RCTs in which the compliance rate was less than 50% [14, 31]. Suboptimal compliance and delays in initiating treatment could possibly diminish the effect of eradicating potential micrometastasis. On the other hand, patients with a better physical condition before surgery were more willing to undergo systemic chemotherapy [14]. With earlier and neoadjuvant delivery of consolidation chemotherapy, SCRT/CCT improved DFS by reducing distant relapse to some extent without compromising local control.

Due to obvious heterogeneity, we adopted a random-effect model when evaluating acute toxicity and observed no statistical differences between groups. If we employed a fixed-effect model, the SRCT/CCT group was inferior to LCCRT groups. The addition of chemotherapy to preoperative treatment possibly resulted in higher toxicity, which was in line with the conclusion from a phase II trial that randomized patients to chemoradiotherapy and surgery with or without FOLFOX induction therapy [32]. Despite considerable acute toxicity during preoperative therapy in the SCRT/CCT group, there were no significant differences noted in the surgical procedures performed or postoperative complications between the two treatment arms. The late complications in the SCRT/CCT group, in our pooled analysis, were inferior with borderline significance (P = 0.05). A long-term follow-up study also showed that patients receiving SCRT, compared to nonirradiated patients, had more postoperative hospitalization due to bowel obstructions and other gastrointestinal complications [33]. It is difficult to indicate that SCRT results in long-term morbidity, as late toxicity is less studied in conventional CRT. To the best of our knowledge, a few randomized trials have demonstrated comparable incidents of late complications between the two treatments during the 3–5 years follow-up period [1, 34].

The addition of consolidation or induction chemotherapy to concomitant neoadjuvant chemoradiation is both safe and effective, as suggested by some small Phase II studies [35, 36]. Such neoadjuvant therapy strategy, compared to LCCRT, not only increased pCR rate, but also improved survival rate [37, 38], which were similar to our results. It is hard to draw a conclusion whether the addition of neoadjuvant chemotherapy to SCRT or LCCRT is better, as less studies are implemented, making a direct comparison difficult. One thing that is certain is that the delivery of five radiotherapy fractions instead of 25 or 28 fractions not only made short-course radiotherapy cheaper and more convenient than conventional concomitant chemoradiation but also decreased the number of treatment days spent in the medical center, especially in the context of the COVID-19 pandemic. This reduction in time spent in the hospital minimized the risk of COVID-19 infection in these susceptible patients [39].

One of the major limitations of this meta-analysis was that 7 included studies contained 3 non-RCTs. Nonetheless, all included studies were of high quality in accordance with the Newcastle–Ottawa criteria or the Cochrane Collaboration’s risk for bias assessment tool. In addition, survival events were calculated at fixed time points as the cumulative survival rate were available only in three studies (two RCTs and one prospective studies). The pooled HR for DFS, again favoring SCRT/CCT, was 0.83 [95% CI (0.70, 0.97), P = 0.02; I^2^ = 38%, fixed model; Additional file 1: Fig. 13], while those for OS was similar between two groups [HR = 0.90, 95% CI (0.74, 1.09), P = 0.27; I^2^ = 0%, fixed model; Additional file 1: Fig. 14]. Last but not least, the cycles of consolidation chemotherapy varied widely in the SCRT/CCT groups, and the optimal regimen for consolidation chemotherapy is still uncertain.

## Conclusion

In summary, with similar OS, surgical procedures and toxicity but improved DFS and pathological outcomes, SCRT/CCT is a rational alternative neoadjuvant treatment for locally advanced rectal cancer, especially in the context of the COVID-19 pandemic.

## Supplementary Information

**Additional file 1.** **Fig. 1**: Risk of bias graph. **Fig. 2**: Risk of bias summary. **Table 1**: Scores of 4 Cohort Studies Using Newcastle-Ottawa Criteria. **Table 2**: Patients characteristics of included studies. **Fig. 3**: Forest plot for downstaging rate. **Fig. 4**: Forest plot for adjuvant therapy pathologic tumor stage 3-4. **Fig. 5**: Forest plot for adjuvant therapy pathologic lymph node positive. **Fig. 6**: Forest plot for local recurrence. **Fig. 7**: Forest plot for distant metastasis. **Fig. 8**: Forest plot for acute toxicity. **Fig. 9**: Forest plot for postoperative complications. **Fig. 10**: Forest plot for late complications. **Fig. 11**: Forest plot for R0 resection rate. **Fig. 12**: Forest plot for sphincter preservation rate. **Fig. 13**: Forest plot for disease-free survival. **Fig. 14**: Forest plot for overall survival.

## Data Availability

All data generated or analyzed during this study are included in this manuscript.

## Abbreviations

LARC: Locally advanced rectal cancer
CRT: Conventionally chemoradiotherapy
SCRT: Short-course radiotherapy
pCR: Pathologic complete response
SCRT/CCT: Short-course radiotherapy followed by consolidation chemotherapy
LCCRT: Conventional long-course chemoradiotherapy
ypTNM: Adjuvant therapy pathologic stage
LR: Local recurrence
DM: Distant metastasis
OS: Overall survival rate
DFS: Disease free survival rate
MESH: Medical Subject Heading
RCTs: Randomized controlled trials
RR: Risk ratio
CI: Confidence intervals
