# Supplementary material for: Preoperative short-course radiotherapy followed by consolidation chemotherapy for treatment with locally advanced rectal cancer: a meta-analysis
Source: Radiat Oncol. 2022 Jan 24;17:14. doi: 10.1186/s13014-021-01974-4 (PMC8785003; doi:10.1186/s13014-021-01974-4)
Supplement: Supplementary file 1 — Additional file 1. Fig. 1: Risk of bias graph. Fig. 2: Risk of bias summary. Table 1: Scores of 4 Cohort Studies Using Newcastle-Ottawa Criteria. Table 2: Patients characteristics of included studies. Fig. 3: Forest plot for downstaging rate. Fig. 4: Forest plot for adjuvant therapy pathologic tumor stage 3-4. Fig. 5: Forest plot for adjuvant therapy pathologic lymph node positive. Fig. 6: Forest plot for local recurrence. Fig. 7: Forest plot for distant metastasis. Fig. 8: Forest plot for acute toxicity. Fig. 9: Forest plot for postoperative complications. Fig. 10: Forest plot for late complications. Fig. 11: Forest plot for R0 resection rate. Fig. 12: Forest plot for sphincter preservation rate. Fig. 13: Forest plot for disease-free survival. Fig. 14: Forest plot for overall survival. [file 13014_2021_1974_MOESM1_ESM.docx]

**Supplementary tables**

**Supplementary table 1: Scores of 4 Cohort Studies Using Newcastle-Ottawa Criteria**

| **Study** | **Selection** | | | | **Comparability** | **Outcome** | | | **Total** |
| --- | --- | --- | --- | --- | --- | --- | --- | --- | --- |
|  | Representativeness of the exposed cohort | Selection of the non-exposed cohort | Ascertainment of exposure | Demonstration that outcome of interest was not present at the start of the study | Comparability of cohorts on the basis of the design or analysis | Assessment of outcome | Was follow-up long enough for outcomes to occur | Adequacy of the follow-up of cohorts |  |
| Markovina  2017 | 1 | 1 | 1 | 1 | 2 | 1 | 1 | 1 | 9 |
| Chung  2017 | 1 | 1 | 1 | 1 | 1 | 1 | 0 | 0 | 6 |
| Thakur  2020 | 1 | 1 | 1 | 1 | 2 | 1 | 0 | 0 | 7 |

**Supplementary table 2: patients characteristics of included studies**

| **STUDY** | **MEAN AGE** | | | **GENDER (MALE)** | | | **STAGE*** | **LOCATION (LOW RECTUM)** | | | **ADJUVANT CHEMOTHERAPY** | | |
| --- | --- | --- | --- | --- | --- | --- | --- | --- | --- | --- | --- | --- | --- |
|  | SCRT/CCT | LCRT | P value | SCRT/CCT | LCRT | P value |  | SCRT/CCT | LCRT | P value | SCRT/CCT | LCRT | P value |
| Markovina  2017 | 57.2 | 56.6 | 0.66 | 71% | 67% | 0.71 | cT3-4N0-2 | 29% | 37.7% | 0.24 | 94.2% | 100% | N/A |
| Chung  2017 | 72 | 72 | 0.76 | 52.6% | 71.3% | 0.16 | cT3-4N0/(+) | 63.2% | 34% | 0.03 | 100% | 100% | 0.22 |
| Ciseł  2019 | 60 | 60 | N/A | 70% | 67% | 0.83 | cT3-4 | 57% | 55% | N/A | N/A | N/A | N/A |
| Bahadoer  2021 | 62 | 62 | N/A | 65% | 69% | N/A | cT2-4N0-2 | 22% | 26% | N/A | 0% | 77% | N/A |
| Thakur  2020 | N/A | N/A | N/A | N/A | N/A | N/A | II-III | N/A | N/A | N/A | N/A | N/A | N/A |
| Aghili  2020 | 56 | 53 | 0.14 | 54.5% | 63% | 0.35 | II-III | 0 | 0 | 0.8 | N/A | N/A | N/A |
| Chakrabarti  2021 | 42 | 43 | N/A | 66.7% | 66% | N/A | II-III | 74% | 80% | N/A | 100% | 100% | N/A |
| Abbreviations: SCRT-CCT, short-course radiotherapy followed by consolidation chemotherapy; LCCRT, long-course chemoradiotherapy; N/A, not available. **STAGE***: clinical stage before treatment. | | | | | | | | | | | | | |

**Supplementary figures**

**Supplementary figure 1: risk of bias graph**
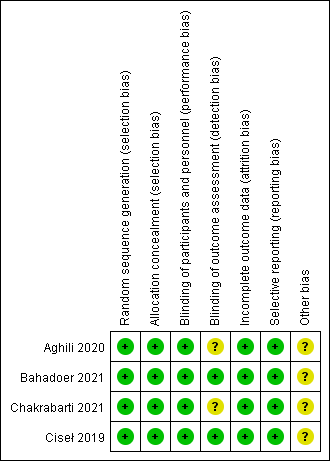


**Supplementary figure 2: risk of bias summary**
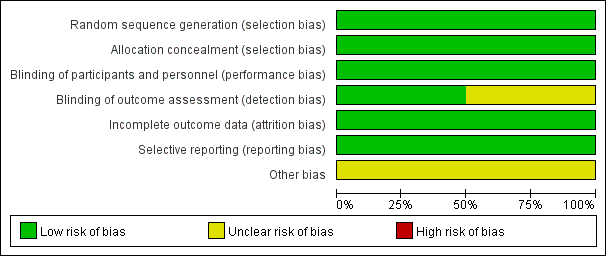


**Supplementary figure 3: forest plot for downstaging rate**


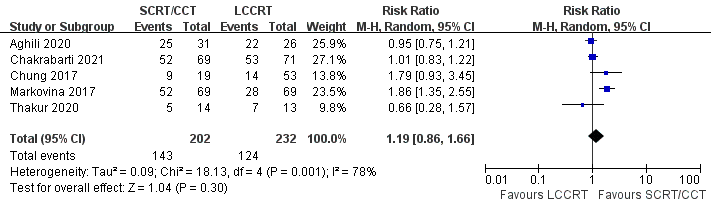


**Supplementary figure 4: forest plot for adjuvant therapy pathologic tumor stage 3-4**
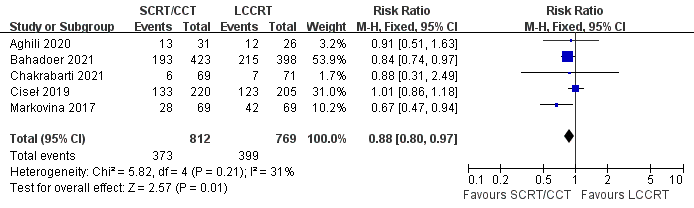


**Supplementary figure 5: forest plot for adjuvant therapy pathologic lymph node positive**
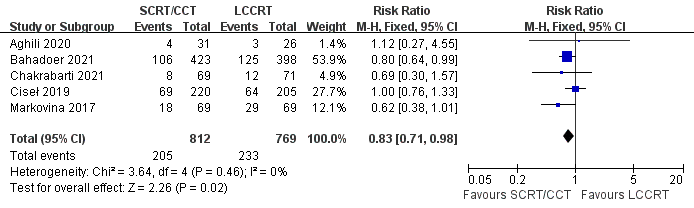


**Supplementary figure 6: forest plot for local recurrence**
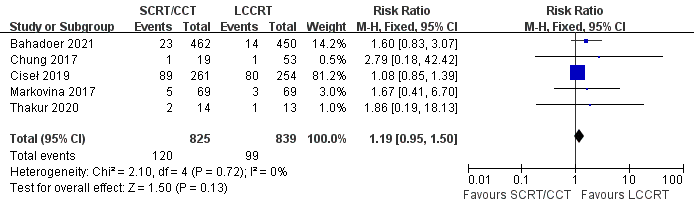


**Supplementary figure 7: forest plot for distant metastasis**


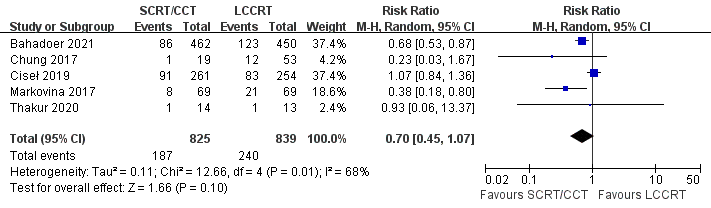


**Supplementary figure 8: forest plot for acute toxicity**
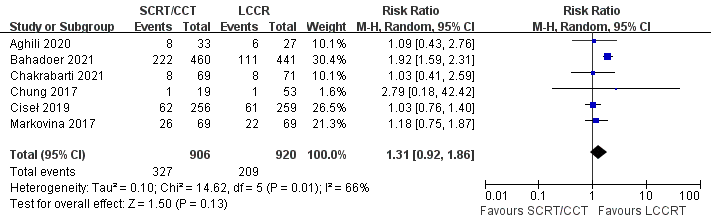


**Supplementary figure 9: forest plot for postoperative complications**


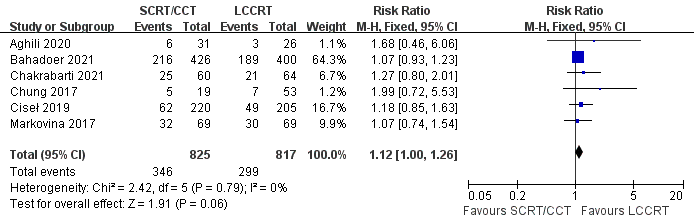


**Supplementary figure 10: forest plot for late complications.**
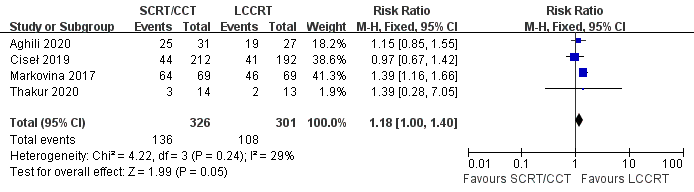


**Supplementary figure 11: forest plot for R0 resection rate.**
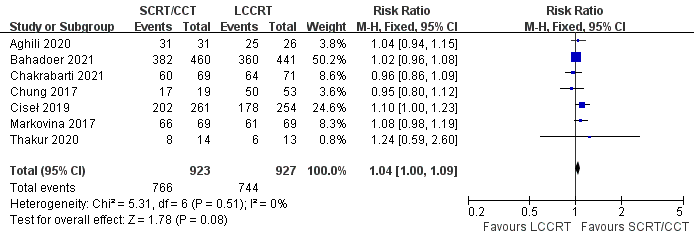


**Supplementary figure 12: forest plot for sphincter preservation rate.**
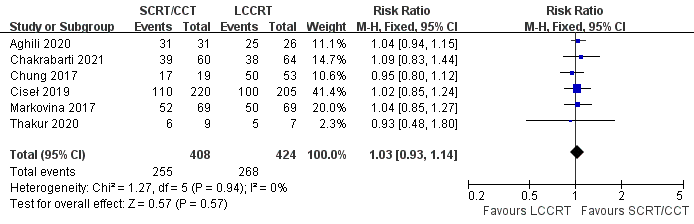


**Supplementary figure 13: forest plot for disease-free survival.**


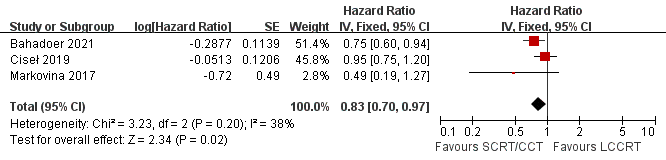


**Supplementary figure 14: forest plot for overall survival.**


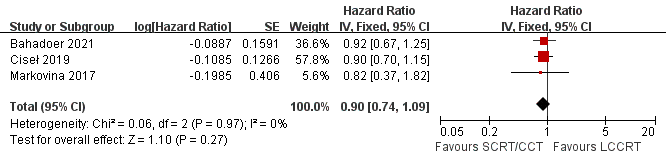


**Detailed preoperative treatment regimens:**

Washington cohort, 2017:

SCRT/CCT group: SCRT (5 Gy x 5 days)→ 9-12 days interval→ mFOLFOX-6 (4 cycles in 6 weeks)→ 4-9 weeks interval→ Surgery→ interval→ Adjuvant chemotherapy (4-6 cycles)；

LCCRT group: LCRT (5-6 weeks)→ 6-8 weeks interval→ Surgery→ interval→ Adjuvant chemotherapy (4-6 cycles).

Polish cohort, 2019:

SCRT/CCT group: SCRT (5 Gy x 5 days)→ 1 week interval→ FOLFOX4 (3 cycles in 5 weeks)→ 6 weeks interval→ Surgery；

LCCRT group: LCRT (6 weeks)→ 6 weeks interval→ Surgery.

Iranian cohort, 2020:

SCRT/CCT group: SCRT (5 Gy x 5-7 days) + XELOX (1 cycle)→ 3-4 weeks interval→ XELOX (3-4 cycles in 9-12 weeks)→ 3-4 weeks interval→ Surgery；

LCCRT group: LCRT (5-5.5 weeks)→ 3-4 weeks interval→ XELOX (3-4 cycles in 9-12 weeks)→ 3-4 weeks interval→ Surgery.

RAPIDO trail, 2020:

SCRT/CCT group: SCRT (5 Gy x 5-8 days)→ 2-4 weeks interval→ CapeOX (6 cycles in 18 weeks) or FOLFOX4 (9 cycles in 16 weeks)→ 2-4 weeks interval→ Surgery；

LCCRT group: LCRT (5-6 weeks)→ 6-10 weeks interval→ Surgery→ Adjuvant chemotherapy (based on center’s preferences).

Indian cohort, 2020:

SCRT/CCT group: SCRT (5 Gy x 5 days)→ 1 week interval→ CapeOX (2 cycles in 6 weeks)→ 4-6 weeks interval→ Surgery→ 4 weeks interval→ Adjuvant chemotherapy；

LCRT (5-6 weeks)→ 4-6 weeks interval→ Surgery→ 4 weeks interval→ Adjuvant chemotherapy.

Korean cohort, 2017:

SCRT/CCT group: SCRT (5 Gy x 5 days) + concurrent chemotherapy→ 8 weeks interval (3 cycles of chemotherapy comprised of leucovorin 400 mg/m² on day 1 and 5-Fluouracil 1200 mg/m² on days 1 and 2 at fortnightly intervals)→ surgery→ 4 weeks interval→ Adjuvant chemotherapy;

LCCRT group: LCRT (6 weeks)→ 8 weeks interval→ surgery→ 4 weeks interval→ Adjuvant chemotherapy.Indian cohort, 2021:

SCRT/CCT group: SCRT (5 Gy x 5 days)→ 6-8 weeks interval (2 cycles XELOX)→ surgery;

LCCRT group: LCRT (6 weeks)→ 8-12 weeks interval→ surgery.
